# Birth preparedness and complication readiness among recently delivered women in Hargeisa town, Somaliland: A community-based cross-sectional study

**DOI:** 10.1371/journal.pone.0302168

**Published:** 2024-04-18

**Authors:** Abdeta Muktar Ahmed, Mohamed Abdilahi Ahmed, Mohammed Hassen Ahmed

**Affiliations:** 1 Department of Public Health, Addis Ababa Medical University College, Hargeisa Campus, Hargeisa, Somaliland; 2 Department of Nutrition, Addis Ababa Medical University College, Hargeisa Campus, Hargeisa, Somaliland; Ethiopian Public Health Institute, ETHIOPIA

## Abstract

**Background:**

About 287,000 mothers lost their lives due to pregnancy and delivery in 2020 worldwide. Birth preparedness and complication readiness (BPCR) is an approach used to utilize the timely use of skilled maternal and neonatal services. Preparing mothers for childbirth and against its dangers has great importance in reducing maternal mortality. Little is known about BPCR and influencing factors in Hargeisa town, Somaliland.

**Objective:**

To assess the level of BPCR and its associated factors among recently delivered women in Hargeisa.

**Methods:**

A community-based cross-sectional study was carried out in September 2022 among 300 women who delivered in the one-year time interval before the study period. A census was done to identify the women, and then they were selected by simple random sampling. Face-to-face interviews were conducted using a pre-structured questionnaire. A woman was considered prepared for birth if she made preparations for at least three of the BPCR components. Data were cleaned, entered, and analyzed using SPSS V.25. Bivariate and multivariate logistic regression analyses were performed with a cut-point of 0.05 significance level.

**Results:**

From a total of 300 women, 38.3% had good knowledge of BPCR, and only one-fourth (25%) were prepared for birth and its complications. Access and media usage (AOR = 9.64, CI 1.09–82.248), receiving health education about BPCR (AOR = 3.75, CI 1.01–13.87), giving birth at health institutions (AOR = 6.02, CI 1.39–25.95), and good knowledge of key danger signs of pregnancy (AOR = 0.017, CI 0.004–0.069) were factors significantly associated with BPCR practice.

**Conclusion:**

The study identified that the BPCR level was very low compared to many other studies. Such a low BPCR level may have a negative impact on maternal health and lives, hindering interventions conducted to reduce maternal mortality rates. All concerned bodies should consider the importance of awareness creation regarding BPCR in their core interventions.

## Introduction

Birth preparedness and complication readiness (BPCR) is an all-inclusive approach intended to promote well-timed utilization of skilled maternal and child health care. It is a planned means to deal with the three delays (delay in seeking care, in reaching care, and in receiving care) at various stages [1]. It is among the important strategies that the World Health Organization (WHO) recommended and included in the focused ANC (antenatal care) provision [2].

Pregnancy as well as delivery-associated complications are the main public health challenges for many nations around the globe [3]. Around two hundred eighty-seven thousand mothers lost their lives due to pregnancy, delivery, and their consequences in 2020 worldwide. The vast majority (95%) of these losses of life happened in low and lower-middle-income nations [4]. According to the first ever Somaliland Health and Demographic Survey (SLHDS) 2020 report, the ANC coverage of the Somaliland was about 47%, and the maternal mortality ratio (MMR) was estimated at 396 maternal deaths per 100,000 live births [5].

Among the leading causes of death among women of reproductive age in developing nations are obstetric-related complications [6]. Many of these dangerous complications are difficult to predict. But they can be prevented with well-timed decisions, preparations, and access to emergency maternal health services [7]. The main reasons for such deaths were frequently attributed to a lack of utilization of available obstetrics care facilities during pregnancy and a lack of adequate planning and preparation for possible dangers related to child delivery. Utilization of existing obstetrics care facilities and having better plans and preparations for possible dangers during pregnancy and childbirth could markedly improve and promote maternal health [8]. Preparing mothers throughout pregnancy for labor and childbirth dangers that can occur at any point in time has paramount importance in reducing maternal mortality and morbidity [3].

BPCR is an approach used to maintain the timely use of skilled maternal and neonatal services, particularly during childbirth, based on the theory that being prepared for delivery and being ready for dangers decrease delay in getting these services [1].

Birth preparedness comprises identifying a skilled provider, having the required plans to obtain skilled care for childbirth, and getting prepared for the safest delivery. It encourages mothers, families, and society to undertake measures such as identifying obtainable transport, preparing money to pay for various fees, and preparing blood donors, thereby enabling quick decision-making and minimizing postponement of getting the services when problems occur. Furthermore, it invites providers and health institutions to get prepared for childbirth and manage obstetric emergencies [1]. It is a major, practically compelling, and rational way of dealing with life-threatening delays. It is an approach providing proper service for the mothers and the neonates during pregnancy, delivery, and postnatal periods, comprising plans for emergency management and setting enabling conditions for the survival of the mothers and neonates [9].

Maternal health is one of the sub-goals included in Goal 3 of the SDG (Sustainable Development Goals). It states that by 2030, the global maternal mortality ratio should be reduced to less than 70 per 100,000 live births, and no country should have a MMR greater than 140 per 100,000 live births [10]. Even though the maternal mortality rate in Somaliland of 396 doesn’t look very high, it is so far from achieving the desired SDG goal of 70 by the year 2030.

In spite of the fact that BPCR strategies are extremely important for the betterment of maternal and child health and the prevention of loss of life, little is known about the status of BPCR and influencing factors among women in Hargeisa town, Somaliland. No studies conducted in Hargeisa, Somaliland, have examined BPCR. Thus, there is a need to understand the level of BPCR and the factors associated with it, with a view to suggesting measures for reducing maternal morbidity and mortality rates. The Somaliland Health and Demographic Survey 2020 (HDS), released in October 2020 [5], didn’t address anything regarding BPCR. Therefore, this study is aimed at assessing the status of BPCR and its associated factors among women who gave birth recently in Hargeisa town, Somaliland.

## Materials and methods

### Study area, period, and design

A community-based cross-sectional study was carried out among 300 women who gave birth in the one-year time interval before the study period in Hargeisa town, Somaliland. The data was collected from September 3 to 29, 2022.

Somaliland is a de facto independent state in the Horn of Africa and broke away from Somalia in 1991. Somaliland is a homogenous country with a population of 4.3 million as of the 2022 report [11]. Hargeisa is the capital of Somaliland, with a population of 1.2 million [12]. The Hargeisa Municipality is divided into six districts, which contain various sub-districts. According to information from the Somaliland Ministry of Health Development, there are 22 private hospitals and 5 public hospitals in Hargeisa. Moreover, there are 25 Maternal and Child Health Centers (MCHs) operating in the town as of 2023 [13].

### Study population

The study population was sampled from all women who gave birth at least once within the one-year period before the study period in Hargeisa town. Women who were seriously ill were not included in the study.

### Sample size and sampling technique

A final sample size of 300 was calculated using a single population proportion formula. A BPCR level of 23.3%, which was taken from a study in Ethiopia, was used [14], with a 5% level of significance, a 5% margin of error, and a 10% non-response rate.

All six districts of Hargeisa town were included in the study. Prior to the survey, a census was done in each district to identify mothers who gave birth within the last 12 months. A total of 1198 women who fulfilled the inclusion criteria were identified and used as a sampling frame. The total sample size was divided proportionally by the number of mothers identified in each district. Codes were given for the identified mothers in their households, and simple random sampling was employed to select, approach, and interview each study subject in each district.

### Data collection instrument and procedure

Face-to-face interviews were conducted by data collectors using a pre-structured, pretested questionnaire that was adapted from JHPIEGO BPCR tools and indicators of maternal and newborn health [1]. The questionnaire contained sociodemographic characteristics, reproductive and obstetrics-related factors, service utilization, knowledge of key obstetrics danger signs, and knowledge and practice of BPCR. The questionnaire was initially prepared in English and then translated into the local Somali language and retranslated back to English by another person to check for any inconsistencies. Accordingly, the necessary modifications were made.

A woman was considered to have good knowledge of key danger signs of pregnancy if she could mention at least two of these key danger signs of pregnancy (vaginal bleeding, swollen hands or face, and blurred vision) spontaneously [8]. A woman was considered to have good knowledge of key danger signs of childbirth if she could mention at least three of these key danger signs (sever vaginal bleeding, prolonged labor (>12 hours), convulsion, and retained placenta) spontaneously [8]. A woman was considered to have good knowledge of key danger signs of postpartum if she could mention at least two of these key danger signs (sever vaginal bleeding, foul-smelling vaginal discharge, and high fever) spontaneously [8].

A woman was considered prepared for birth and its complications if she had made preparations for at least three of the BPCR components (identified place of delivery, identified skilled health care provider, saved money, identified transport ahead of emergency, and identified blood donor) [3, 8]. A woman was considered to have good knowledge of BPCR if she mentioned at least three of the five BPCR components. The dependent variable, BPCR, was later dichotomized as “**prepared”** and “**not prepared”** for delivery and its complications. Independent variables included socio-demographic variables (such as age, marital status, education, occupation, income, and media usage), obstetrics-related variables (such as number of pregnancies and births, history of stillbirth and abortion, BPCR knowledge), service utilization variables (such as ANC utilization, health education given, timing of ANC, place of birth, decision-making), and knowledge of key obstetric danger signs.

### Data processing and analysis

After data collection, each questionnaire was checked for completeness, clarity, and consistency. Data was entered and analyzed using SPSS (Statistical Package for Social Studies) version 25. Bivariate analysis was performed to determine the existence of association between dependent and independent variables. Then variables with a p-value of less than 0.2 in bivariate analysis were included in a single model, and multiple logistic regressions were performed. The strength of the statistical association was measured by the AOR (adjusted odds ratio) at 95% CI (confidence interval). Statistical significance was declared at a p-value of less than 0.05.

### Data quality control

To assure the quality of the data, a data collection tool was prepared after an intensive review of relevant literature and related studies. Initially, the questionnaire was prepared in English, then translated to Somali and back to English by different individuals with good ability in both languages. Training was given to both data collectors and supervisors by the principal investigators. Pre-testing of the questionnaire was carried out on 30 women (10% of the sample size) who were not included in the study (in a nearby town, Gabiley). Accordingly, the necessary modifications were made. The collected data was checked for completeness before data entry.

### Ethical consideration

The study obtained ethical clearance from the Research Ethics Committee of Addis Ababa Medical University College (AAMUC), Hargeisa campus (Ref No.: AAMUCHC/EC/90/2022). All methods and procedures performed in the study followed the relevant ethical standards and guidelines of the university and the Helsinki Declaration. For all participants, the aim of the study was explained, and they were reassured that their responses would only be used for research purposes and would remain confidential. Similarly, after a clear discussion about the purpose of the study, written informed consent was obtained from each study subject, while the study subjects’ right to refuse was also respected. To assure the confidentiality of the study subjects’ responses, writing their names or any identification in the questionnaire was not required.

## Results

### Sociodemographic characteristics of the respondents

A total of 300 women who gave birth in the past 12 months preceding this study participated in the study. Their age ranged from 18 to 43 years, with a mean (±SD) of 28.9 years (±5.8) and a median of 29, of which more than half, 172 women (57.3%), belonged to 25–34 years. The mean age of the first marriage was 20.6 years, with a minimum of 15 and a maximum of 28. The great majority, 259 women (86.3%), were living permanently in Hargeisa; 275 (91.7%) were married; 182 (60.7%) had primary or secondary education; 171 (57%) were merchants and employed; and 179 of their spouses (65.7%) were employed. Two hundred and thirty-two women (77.3%) had access to and usage of media. The mean monthly income as reported by the respondents was 513 US dollars, of which 134 women (44.7%) had an income range of 150–300 (Table 1).

**Table 1:** Socio-demographic characteristics of respondents, Hargeisa, Somaliland, September 2022.

Variable	Response	Frequency (n = 300)	%
Age	18–24	71	23.7
	25–34	172	57.3
	35–43	57	19
Permanent address	In Hargeisa	259	86.3
	Outside Hargeisa	41	13.7
Marital status	Married	275	91.7
	Widowed	13	4.3
	Divorced	12	4
Educational status	No formal education	46	15.3
	Primary education	95	31.7
	Secondary education	87	29
	Tertiary education	72	24
Age at first marriage	15–17	45	15
	18–24	214	71.3
	25–28	41	13.7
Access and usage of media	Yes	232	77.3
	No	68	22.7
Monthly income in US dollars	≤300	134	44.7
	>300	166	55.3
Occupation	Housewife/no job	111	37
	Employed	74	24.7
	Merchant	97	32.3
	Daily laborer	18	6
Spouse employment	Employed	197	65.7
	Not employed	103	34.3

### Obstetric characteristics of the respondents

Only 37.3% of the respondents (112 women) had ever heard the term BPCR, mainly from health professionals and the media (89.3%). Two hundred and forty-eight women (82.7%) had at least one ANC visit in their lifetime. Thirty-nine women (13%) among all respondents reported they had a history of stillbirth, while 91 (30.3%) had abortions. The women had a mean of 3.4 pregnancies and 3.06 deliveries. More than half, 151 women (53.7%), reported that they had independent decision-making power regarding obstetrics service utilization. The majority of the women, 181 (60.3%), gave their last birth at health institutions (Table 2).

**Table 2:** Obstetric characteristics of respondents, Hargeisa, September 2022.

Variable	Response	Frequency (n = 300)	%
Ever heard about BPCR	Yes	112	37.3
	No	188	62.7
Ever had ANC	Yes	248	82.7
	No	52	17.3
Any stillborn birth ever	Yes	39	13
	No	261	87
Any abortion ever	Yes	91	30.3
	No	209	69.7
Number of total pregnancies	1	59	19.7
	2–4	170	56.7
	5 and more	71	23.6
Number of total deliveries	1	63	21
	2–4	176	58.7
	5 and more	61	20.3
Decision-making regarding obstetrics service utilization	The women only	161	53.7
	The spouse only	52	17.3
	Both	83	27.7
Place of birth	Home	119	39.7
	Health institution	181	60.3
Place of birth planned ahead	Yes	264	88
	No	36	22

Of the 248 women who had at least one ANC visit in their lifetime, the majority, 191 (77%), had an ANC visit during their last pregnancy. From these 248 women, only 97 women (39.1%) reported they received advice and health education regarding BPCR during their ANC visits, and 36 (14.5%) reported they were accompanied by their spouses. Among the 191 women who had their ANC visits during their last pregnancy, 76 women (39.8%) had their first visit during the second trimester, while the remaining 63 (33%) and 52 (27.2%) had them during the first and third trimesters, respectively (Table 3).

**Table 3:** ANC-related characteristics, Hargeisa, September 2022.

Variable	Response	Frequency (n = 248)	%
Advice about BPCR ever	Yes	97	39.1
	No	151	60.9
Accompanied by spouse ever	Yes	36	14.5
	No	212	85.5
ANC visit during the last pregnancy	Yes	191	77
	No	57	23

### Knowledge of key obstetric danger signs

The women mentioned as many key danger signs of pregnancy, childbirth, and postnatal periods as possible. Vaginal bleeding was the top-listed key danger sign of pregnancy, mentioned by 77.7% of the women. It was similarly mentioned as the top key danger sign of childbirth and postnatal periods by 66.3% and 65.3%, respectively. Accordingly, 106 women (35.3%), 84 (28%), and 118 (39.3%) had good knowledge of key danger signs of pregnancy, labor and childbirth, and the postnatal period, respectively (Table 4).

**Table 4:** Knowledge level and key obstetrics danger signs mentioned by the respondents, Hargeisa, Somaliland, September 2022.

Obstetric danger signs mentioned	Variables	Frequency (%)
During pregnancy	Vaginal bleeding	233 (77.7%)
	Blurred vision	101 (33.7%)
	Swollen faces and limbs	85 (28.3%)
During childbirth	Vaginal bleeding	199 (66.3)
	Retained placenta	157 (52.3%)
	Prolonged labor (>12 hours)	145 (48.3%)
	Convulsion	50 (16.7%)
During postpartum	Vaginal bleeding	196 (65.3%)
	Foul-smelling vaginal discharge	161 (53.7%)
	High fever	77 (25.7%)
Knowledge level of key obstetrics danger signs	During pregnancy	106 (35.3%)
	During labor and childbirth	84 (28%)
	During postnatal	118 (39.3%)

### Knowledge and practice of BPCR

**Fig 1:**
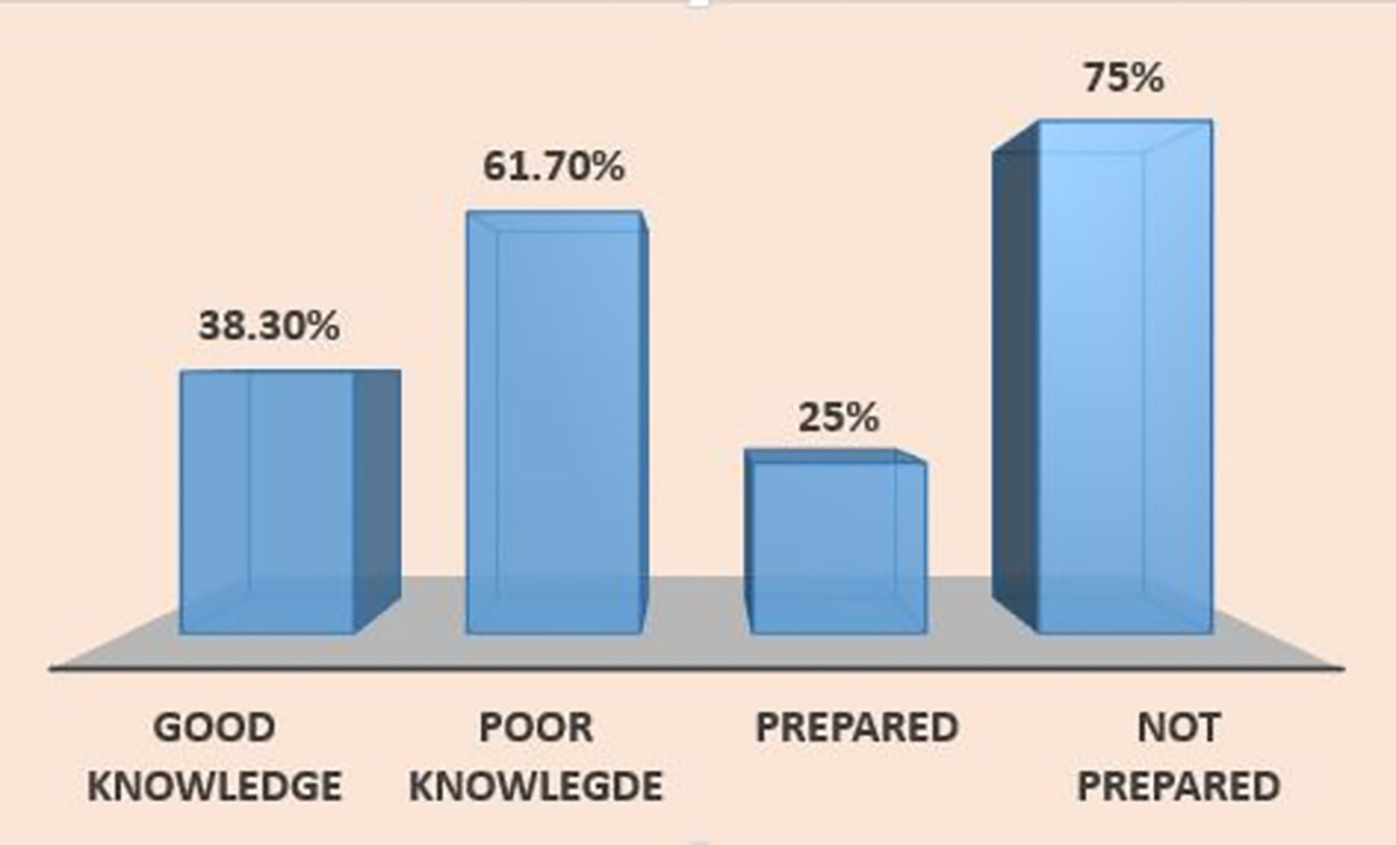
Status of BPCR of respondents, Hargeisa, Somaliland, September 2022.

Regarding knowledge of BPCR, 115 of the respondents (38.3%) had a good knowledge of BPCR (mentioned at least 3 of the 5 components). On the other hand, only 75 women (25%) were prepared for birth and its potential complications (Fig 1).

Respondents who mentioned “saving money” accounted for 210 (70%). One hundred and sixty-one women (53.7%) mentioned “identifying place of delivery,” whereas the other 120 (40%) and 99 (33%) mentioned “identifying transportation” and “identifying skilled providers,” respectively. “Preparing blood donors” was mentioned only by 91 women (30.3%).

Regarding BPCR practice, 254 women (85%) prepared essential items for clean delivery and the post-partum period; 203 women (67.7%) saved money; 136 (45.3%) identified the place of delivery; 83 (27.7%) identified a skilled provider; 76 (25.3%) identified transportation; and the rest, 31 (10.3%), identified blood donors (Table 5).

**Table 5:** Knowledge and practice of BPCR, Hargeisa, Somaliland, September 2022.

Variables	Response	Frequency (%)
Knowledge of BCPR	Preparing essential items for clean delivery	254 (85%)
	Arranging or saving money for delivery	210 (70%)
	Identifying the place of delivery	161 (53.7%)
	Identifying means of transportation	120 (40%)
	Identifying a skilled provider	99 (33%)
	Arranging blood donors	91 (30.3%)
	Designating a decision-maker on her behalf	45 (15%)
	Arranging ways of communication	34 (11.3%)
Practice of BPCR	Prepared essential items for clean delivery	230 (76.7%)
	Arranged or saved money for delivery	203 (67.7%)
	Identified the place of delivery	136 (45.3%)
	Identified a skilled provider	83 (27.7%)
	Identified means of transportation.	76 (25.3%)
	Arranged blood donors	31 (10.3%)
	Arranged way of communication	19 (6.3%)
	Designated decision-maker on her behalf	13 (4.3%)

### Factors associated with BPCR

Bivariate and multivariate analyses were done using logistic regression to analyze factors associated with BPCR and rule out possible confounders. On bivariate analysis, access to media, hearing about BCPR, attending ANC, health education regarding BPCR during ANC, place of delivery, and knowledge of key obstetric danger signs of pregnancy, childbirth, and postpartum were associated with BPCR.

But, on multivariate analysis, access to media, health education regarding BPCR during ANC, place of delivery, and knowledge of key obstetric danger signs of pregnancy were associated with BPCR (Table 6).

**Table 6:** Bivariate and multivariate analysis showing factors associated with BPCR, Hargeisa town, Somaliland, September 2022.

Variables	Status of the BPCR		COR (95% CI) p-value	AOR (95% CI) p-value
	Prepared (%)	Not Prepared (%)		
**Access to and usage of media**				
Yes	73 (97.3)	159 (70.7%)	15.15 (3.612–63.551)* <0.001	9.46 (1.09–82.248)* 0.042
No	2 (2.7%)	66 (29.3%)	1	1
**Health education on BPCR during the ANC**				
Yes	47 (68.1%)	50 (27.9%)	5.5 (3.018–10.068)* <0.001	3.75 (1.01–13.87) * 0.047
No	22 (31.9%)	129 (72.1%)	1	1
**Place of birth**				
At health institution	4 (5.3%)	115 (51.1%)	18.55 (6.555–52.531)* <0.001	6.02 (1.39–25.95)* 0.016
At home	71 (94.7%)	110 (48.9%)	1	1
**Knowledge of key danger signs of pregnancy**				
Poor knowledge	3 (4%)	191 (84.9%)	0.007 (0.002–0.025)* <0.001	0.017 (0.004–0.069)* <0.001
Good knowledge	72 (96%)	34 (15.1%)	1	1

*Statistically significant (P<0.05).

Women who had access to and usage of media (AOR = 9.46, 95% CI 1.09–82.24, P = 0.042) were nine times more likely to get prepared for birth and its complications compared to those who did not have. Women who received health education regarding BPCR during ANC visits (AOR = 3.75, 95% CI 1.01–13.87, P = 0.047) were 3.7 times more likely to get prepared for birth and its complications compared to those who did not receive it. And women who gave their last birth at health institutions (AOR = 6.02, 95% CI 1.39–25.9, P = 0.016) were six times more likely to get prepared for birth and its complications compared to those who gave birth at home. Women who did not have good knowledge of obstetric danger signs of pregnancy (AOR = 0.017, 95% CI 0.004–0.069, P<0.0001) were less likely to get prepared for birth and its complications compared to those who had good knowledge.

## Discussion

Birth preparedness and complication readiness is one of essential elements of antenatal care, whose goal is to decrease any unnecessary delays in seeking and receiving care. It helps pursue emergency obstetrics care and hence improve maternal and fetal outcomes [1]. It is a simple and forward-looking strategy aimed at the early and timely handling of obstetric complications if they arise. No study was done in Hargeisa, Somaliland, regarding BPCR. Thus, the current community-based study focused on determining the level of BPCR practice among recently delivered women and associated factors.

The current study identified that only 37.3% of the respondents had ever heard the term BPCR. This percentage was lower compared to other facility-based studies conducted in Cameroon, in which 46.1% heard the term [15], in Chiro, 89.4% [16], and in Dilchora Hospital, Dire Dawa, 74.7% [17]. This might be due to the fact that the current study was a community-based study in contrast to the facility-based studies in which the study participants had better exposure to maternal health services, which might help them become acquainted with and absorb the term BPCR. On the other hand, in a similar community-based study done in Goba, Ethiopia, 46.4% of study participants heard the term BPCR [18]. The current study still fell behind. Awareness is the first step towards knowledge that may help people become conscious of beneficial information and hence produce desired behavior later.

Among respondents who had ever heard the term BPCR, the current study indicated that 50% of the respondents heard it mainly from health professionals and 39.3% from the media. Thus, health professionals and the media remained the major sources of awareness of BPCR. This is in line with studies done in Goba district [18], which revealed health professionals and the media were major sources of awareness. Similar reports in Ethiopia, Guraghe [8], Chiro [16], and Harari [19] indicated health professionals as the major source of information. In the present study, 14.5% of the study subjects who had ever had ANC reported being accompanied by their spouses. This was lower compared to the similar community-based study conducted in North Nigeria that showed 32.1% were accompanied [20]. Husbands’ involvement in obstetric care, such as during ANC and delivery care, might have paramount importance in creating better awareness, thereby providing the support needed for the early detection and management of complications.

Around one-third of all respondents (35.3%) had good knowledge of key danger signs of pregnancy, which was comparable to the report in Gurage Zone’s 34.9% knowledge level [8]. However, it was relatively higher compared to studies conducted in Kenya (21%) and Tanzania (17.8%) [21], and Addis Ababa (11.1%) [22]. It was lower than a study at Dire Dawa Dilchora Hospital (58.6%) [17]. Likewise, the current study revealed 28% and 39.3% had good knowledge of key danger signs of childbirth and postpartum periods, respectively, which were higher than the study in Addis Ababa [22], in which 14.2% and 10.9% had good knowledge of childbirth and postpartum periods, respectively. The discrepancies might be attributed to variations in the implementation of maternal health services. Sociodemographic factors and disparities in the study site and settings might as well contribute to the inequalities.

Our finding indicated that 38.3% of the women interviewed had good knowledge of BPCR (mentioned at least 3 of the 5 components), which was higher than findings in Addis Ababa (15.2%) [22] and Goba (14.6%) [18]. Though our study seemed to be relatively higher compared to others, it was not absolutely satisfactory, as proper knowledge is the basis for positive and helpful practice.

The exact quarter of the study participants (25%) were well prepared for birth and its potential complications, which was comparable with various community-based studies in Bangladesh at 24.5% [23], Goba at 29.9% [18], Adigrat at 22% [24], Wollo at 24.1% [25], a meta-analysis study in Ethiopia at 25.2% [26], and a facility-based study in Rwanda at 22.3% [27]. On the other side, our finding was comparatively higher than studies conducted in Dodoma, Tanzania, 16.7% [28], in Cameroon, 18.8% [15], in rural Bangladesh, 12.2% [29], in Kenya, 11.4%, and in Tanzania, 7.6% [21], and findings in Ethiopia, Arsi, 16.5% [30], and Wolaita, 18.3% [31]. In contrast, the current study was relatively lower than studies done in Chamwino, Tanzania, 58.2% [32], in India, 47.8% [9], and other studies in Ethiopia, such as in Dire Dawa, 54.7% [17], in Harari region, 52.1% [19], and in Gurage, 37% [8]. The disparities might be related to variations in study settings, sociodemographic features, and differences in strategies and applications of maternal health services in these several regions around the world. Low BPCR practice levels, such as in our study, call for well-organized and coordinated efforts, as it becomes more difficult to manage complications if they arise than to identify, recognize, and prevent them.

Concerning BPCR practice, 67.7% of the respondents saved money, which was one of the top-ranked and practiced components in our report, which was similar to studies in Bangladesh [23], and Chamwino Tanzania [32]. This was probably due to the fact that money can help women deal with obstetric uncertainties and buy items needed for birth and care of the newborn. However, as many as 85% of respondents in our study prepared essential items for clean delivery and post-partum period, such as foods, clean clothing, and other items. Such a high proportion of preparation of items for clean delivery and post-partum, including food, was also reported in findings in Dodoma, Tanzania, 62.9% [28], Goba, 87.6% [18], Gondar, 90.81% [3], and Gurage, 98.4% [8].

On the contrary, arranging potential blood donors was the least practiced BPCR element in our study, by only 10.3% of the participants. Such a low level was also reported in findings in Chamwino, Tanzania, at 17.5% [32] and in Wolaita at 3% [31]. It indicated that the respondents were more prepared for minor items such as clean clothing, foods, and other materials than crucial and basic obstetric preparations, such as arranging blood donors and identifying the place of delivery.

On the other hand, 45.3%, 27.7%, and 25.3% of our study participants identified places of delivery, skilled providers, and transportation, respectively, which were all lower than studies conducted in Chamwino, Tanzania [32], Delhi, India [33], Diredawa [17], and Harari region in Ethiopia [19]. That seemed to be the reason why the level of BPCR was so low in our study compared to most of the other studies we used for comparison.

Regarding factors associated with BPCR, women who had access to media and used it were more likely to get prepared for birth and its complications compared to those who did not. Similar result was also reported in Bangladesh [23]. The implication of the finding is that the media plays a great role, as it was reported as one of the major sources of awareness about BPCR. It might also raise the level of awareness about health matters, thus alerting the listeners.

And women who received health education regarding BPCR were more likely to get prepared compared to those who did not receive it. Likewise, several reports from Ethiopia in Gondar, Adigrat, and Wollo [3, 24, 25] supported this, as information and education are initial and crucial steps to bring about positive behavioral changes. Health professionals were regarded as trusted sources of health and health-related information and could help people make healthier choices.

Place of delivery was one of the predictors of BPCR in our findings. Thus, women who gave birth at health institutions were more likely to be prepared for birth and its complications. Other studies also reported the same finding, such as in Arsi [30], Chamwino Tanzania [32], and Kenya and Tanzania [21]. This could be described by the fact that women who gave birth at health facilities have a better opportunity to get health information on the importance of BPCR, providing them with better choices, empowerment, and decisions. Women could get emergency care as well if dangerous or life-threatening conditions occurred.

Knowledge of obstetric danger signs could help women be well-prepared and equipped for childbirth, boost helpful decision-making power, and promote healthy behavior. In our study, women who had good knowledge of the obstetric danger signs of pregnancy were more likely to get prepared compared to those who had poor knowledge. The same was reported in a number of studies in rural Bangladesh [29], Chamwino Tanzania [32], Harari region [19], Arsi [30], Goba [18], Wolaita [31], and Chiro [16].

## Conclusion

Overall, the findings of this study provided valuable insights into the determinants of the practice of BPCR among recently delivered women in Hargeisa town. The prevalence of BPCR practice was low (25%) compared to many other study findings. The study also identified that a large proportion of the women did not have good knowledge of BPCR. A low BPCR level may have a negative impact on maternal lives and may hinder interventions conducted to reduce maternal mortality and morbidity rates. All concerned bodies should plan and implement various evidence-based strategies, such as awareness creation on BPCR and expanding maternal health service utilization in their core interventions. Training the midwives on the importance of educating mothers about BPCR and empowering women should be considered as well.

### Limitations and strength of the study

#### Limitations

The cross-sectional nature of the study made it impossible to reach a casual relationship between the different independent variables and BPCR practice. The source of information was merely based on the self-reports of respondents. There might be social desirability and recall biases.

#### Strength

We used a community-based study, which could better generalize the findings and provide a real picture of the problem in the community.

With the aforesaid limitations and strengths in mind, we determine that our findings have implications for implementations related to BPCR in the area and beyond.

## Supporting information

S1 File SPSS dataset BPCR. (SAV)
